# Alveolar Bone Ridge Augmentation Using Polymeric Membranes: A Systematic Review and Meta-Analysis

**DOI:** 10.3390/polym13071172

**Published:** 2021-04-06

**Authors:** Manuel Toledano-Osorio, Manuel Toledano, Francisco Javier Manzano-Moreno, Cristina Vallecillo, Marta Vallecillo-Rivas, Alberto Rodriguez-Archilla, Raquel Osorio

**Affiliations:** 1 Colegio Máximo de Cartuja s/n, Faculty of Dentistry, University of Granada, 18071 Granada, Spain; mtoledano@correo.ugr.es (M.T.-O.); cvallecillorivas@hotmail.com (C.V.); mvallecillo@correo.ugr.es (M.V.-R.); alberodr@ugr.es (A.R.-A.); rosorio@ugr.es (R.O.); 2 Medicina Clínica y Salud Pública PhD Programme, University of Granada, 18071 Granada, Spain; 3 Biomedical Group (BIO277), Department of Stomatology, School of Dentistry, University of Granada, 18071 Granada, Spain; fjmanza@ugr.es; 4 Instituto Investigación Biosanitaria, ibs. Granada, 18071 Granada, Spain

**Keywords:** bone regeneration, polymeric membrane, bone substitutes, ridge augmentation

## Abstract

Alveolar bone ridge resorption occurred after natural teeth loss and it can restrict the possibility of dental implants placement. The use of bone regenerative procedures is frequently required. The existing evidence regarding the efficacy of horizontal bone ridge augmentation trough guided bone regeneration (GBR) using polymeric membranes was stated. A systematic review and meta-analysis were performed. Electronic and manual literature searches were conducted. Screening process was done using the National Library of Medicine (MEDLINE by PubMed), Embase, and the Cochrane Oral Health. Included articles were randomized controlled trials and observational studies. Weighted means were calculated. Heterogeneity was determined using Higgins (*I*2). If I2 > 50% a random-effects model was applied. It was found that the mean of horizontal bone gain was 3.95 mm, ranging from 3.19 to 4.70 mm (confidence interval 95%). Heterogeneity is I2 = 99% (confidence interval 95%) and significance of the random-effects model was *p* < 0.001. The complications rate was 8.4% and membrane exposure was the most frequent. Through this study, we were able to conclude that the existing scientific evidence suggests that GBR using polymeric membranes is a predictable technique for achieving horizontal bone augmentation, thus, permitting a proper further implant placement.

## 1. Introduction

Dental implants have become a predictable treatment option therapy after teeth loss. To perform an optimal implant placement and to improve their long-term prognosis, it is frequently required alveolar ridge augmentation to increase bone volume [1]. Alveolar ridge resorption occurred after teeth loss, trauma, or infections and it can severely restrict dental implant placement [2]. The use of bone regenerative procedures is frequently required before implant placement. Alveolar ridge defects may be classified according to the main resorbed region as horizontal, vertical, or combined defects [2]. The loss of horizontal ridge width occurs more frequently and to a greater extent compared with the loss of vertical ridge height [3].

Different approaches may be used to regenerate atrophic alveolar ridges, but guided bone regeneration (GBR) is the most frequently used technique [2,4]. For GBR various biomaterials are applied: (1) autogenous, allogenic, xenogeneic, and synthetic bioceramics of polymers can be use as bone substitute materials and can be particulated or as a block unit [5] and (2) resorbable or non-resorbable polymeric membranes which will act as barriers, playing an important role by isolating soft tissue and allowing bone to grow. Several combinations of materials may be employed but the use of polymeric barrier membranes is highly encouraged as it prevented significant bone resorption during the healing period and, thus, greater mean ridge width gain is obtained [3,6]. The barrier polymeric membrane excluded undesirable cells, for example, epithelial and connective tissue cells from populating the wound site, therefore allowing cells with regenerative potential, for example, osteoblasts to colonize the defect and form bone [7]. Non-resorbable membranes, with polytetrafluroethyelene (PTFE) membranes as the maximum representative, have a superior space-making capability but there is a high frequency of wound dehiscence and the subsequent risk of bacterial contamination and infection that require retreatment [5,8,9]. Trying to avoid this problem, antibiotics and metal such as silver, zinc, or copper have been incorporated into the GBR membranes to improve periodontal healing [10,11,12,13]. Another drawback of these membranes is the need of a second surgery in order to retrieve the membrane, besides, the difficulty of this process due to their soft tissue integration [5]. Meanwhile, resorbable membranes, which are mainly represented by collagen membranes, are mainly limited by their lack of rigidity, reducing the space-making potential [14]. Another downside of this membranes is their fast degradation rate, which may not meet the necessary period for an optimal tissue regeneration [5]. This problem has been partially solved by the chemical cross-linking of the collagen matrices. This enhances the collagen stability, but it has also been associated with severe inflammation at the surgical site due to the release of chemical residues [5]. 

The main objectives of this study were to gather all of the scientific evidence about the effectiveness of GBR achieved with polymeric membranes and to quantify the expectable amount of horizontal bone gain than can be obtained. 

## 2. Materials and Methods

### 2.1. Development of the Protocol 

The study protocol was designed according to the Preferred Reporting Items for Systematic Review and Meta-Analysis (PRISMA) statement. The developed protocol was registered in the PROSPERO - International Prospective Register of Systematic Reviews database hosted by the National Institute for Health Research, University of York, Center for Reviews and Dissemination (ID: CRD42021232447). Details regarding the PICO question (population, intervention, comparison, and outcome) are the following: 

P—Healthy patients, older than 18 years, with a deficient alveolar ridge that needs to be horizontally augmented prior to implant placement. 

I—Guided Bone Regeneration with bone graft materials (resorbable or non-resorbable membranes alone or with the addition of bone graft substitutes as autografts, xenografts, alloplasts, or allografts.

C—Defects pre-treatment and post-treatment (between 3 and 7 months of follow-up) or other surgical approaches. 

O—Bone gain after treatment, measured with cone beam computed tomography (CBCT). Secondary outcomes are clinical benefits and/or biological complications of GBR. 

Articles considered eligible for inclusion were interventional (randomized controlled trials (RCTs)) and observational studies (cohort, case-control studies, and case series). A minimum sample size of 5 patients was required.

Exclusion criteria included: animal studies, in vitro studies, reviews and articles published in a language other than English, studies assessing lateral or vertical bone augmentation or horizontal when in conjunction with immediate implant placement. 

### 2.2. Search Strategy, Data Extraction, and Studies Quality Assessment

Electronic and manual literature searches were conducted by 2 independent reviewers (M.T-O. and R.O.). The following search strategy was followed: (“collagen membrane” OR “extracellular membrane” OR “porcine collagen membrane” OR “porcine derived collagen membrane” OR “cytoplast” OR “PTFE membrane” OR “Bio-guide”) AND (“guide bone regeneration” OR “Bone augmentation” OR “GBR” OR “ridge augmentation”) NOT (“sinus lift” OR “sinus elevation” OR “ridge preservation” OR “socket preservation” OR “animal” OR “dog” OR “pig” OR “rabbit”). A time frame restriction of 15 years was applied. Screening process was performed at the following information sources: the National Library of Medicine (MEDLINE by PubMed), Embase and the Cochrane Oral Health. Databases were searched for studies published up to including January 2021. Reference lists of the previous reviews and included studies were analyzed trying to search for relevant manuscripts that were missing after the electronic screening.

Data extraction and risk-of bias were assessed by two investigators (M.T-O. and C.V.) in duplicate and thereafter discussed to find agreement. In the case of disagreement, the judgment of a third reviewer (R.O.) was decisive. The following data were extracted: (1) authors and year of publication, (2) study design, (3) participants and number of interventions, (4) bone substitute, (5) membrane, (4) follow-up time, (5) bone gain, and (6) clinical complications. 

The study quality and designs were evaluated according to: (i) An adapted version of the Newcastle–Ottawa scale [15] for interventional and observational researches. Studies were considered as having a high, medium, or low methodological quality and (ii) The Joanna Briggs Institute Critical Appraisal tool for case series. Studies were considered as having a high, medium, or low risk of bias [15]. 

### 2.3. Data Analyses

Descriptive statistics were used to present the primary outcome—efficacy of GBR in terms of bone gain (mm). Weighted means (CI 95%) were calculated, including total sample size, inverse variance, and standard error of the treatment effect. Heterogeneity was determined using Higgins (I2). If I2 > 50% a random-effects model was applied. Statistical significance was set at 0.05. Data were analyzed with RevMan 5.4 (The Cochrane Collaboration, Oxford, UK). Funnel plot was produced by MedCalc 18.2.1 (MedCalc Software Ltd., Ostend, Belgium) to represent systematic heterogeneity.

## 3. Results

### 3.1. Search Results 

**Figure 1:**
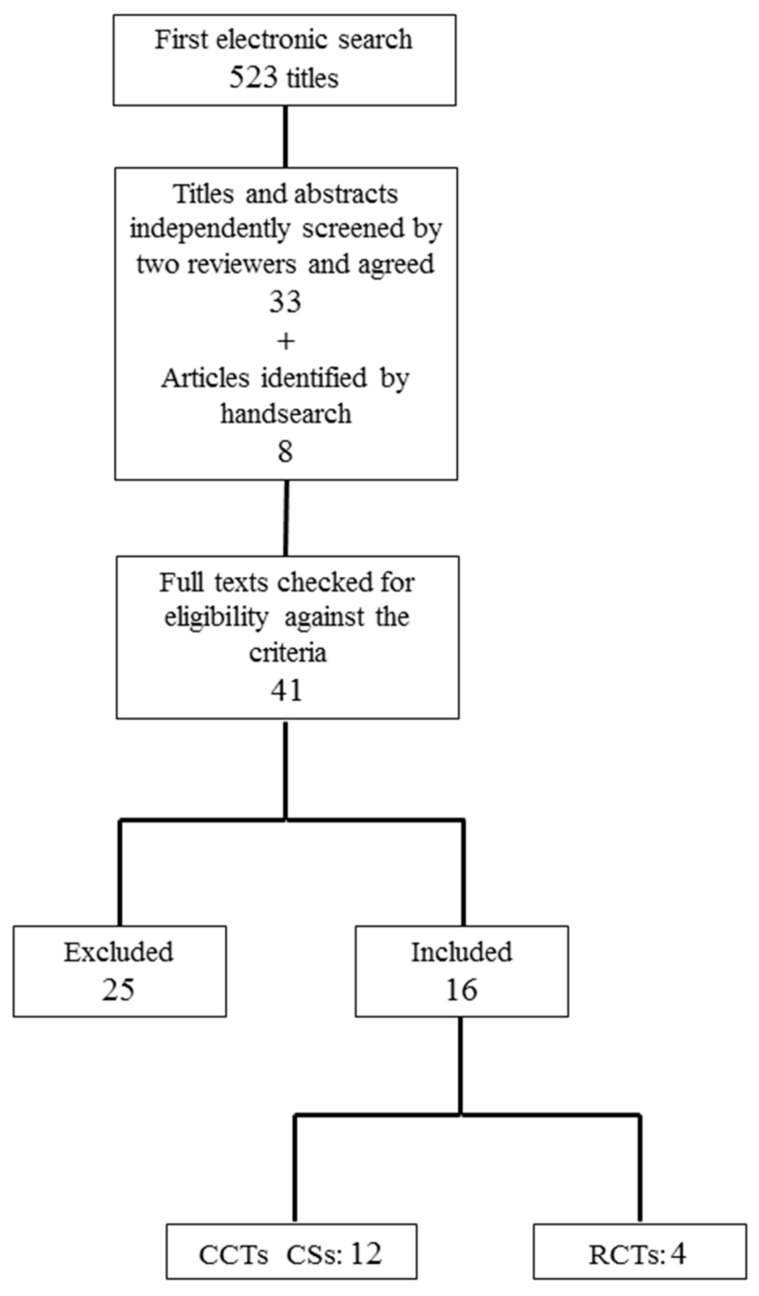
Flow chart of the search results and respective selection process. RCTs: randomized controlled trials, CCTs: cohort and case-control studies, CSs: case series.

The electronic search was performed in January 2021, resulting in 523 articles. After duplicate removal and the reading of titles and/or abstracts, 41 articles were selected. A manual search identified eight more manuscripts. Then the full-text of all the selected articles was reviewed for the inclusion criteria. Then, 25 articles were excluded after full reading, and 16 articles were then included in the final selection. A flowchart of the selection and inclusion process, based on PRISMA recommendations is presented in Figure 1. The extracted data for each reviewed article are shown in Table 1. 

### 3.2. Studies Quality Assessment and Risk of Bias 

**Figure 2:**
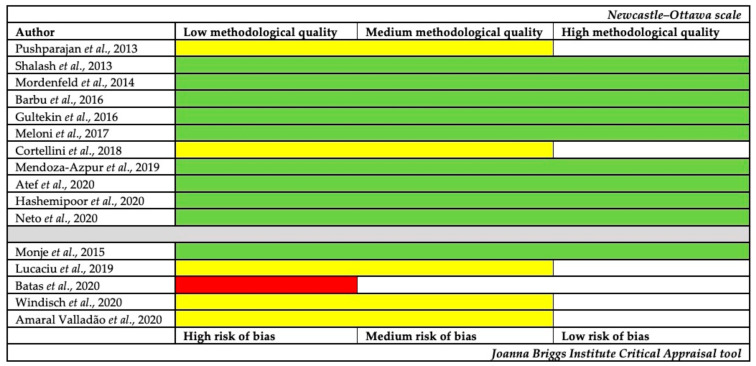
Studies’ quality assessment and risk of bias following: Newcastle–Ottawa scale for interventional and observational assays. Studies were considered as having a high (green), medium (yellow), or low (red) methodological quality, and the Joanna Briggs Institute Critical Appraisal tool for case series. Studies were considered as having high (red), moderate (yellow), or low (green) risk of bias.

The quality assessment and the risk of bias of the selected papers are summarized in Figure 2. Most of the selected studies are classified as high quality or low risk of bias. 

### 3.3. Primary and Secondary Outcomes: Horizontal Bone Gain and Complications

Sixteen studies (292 patients and 381 defects) analyzed the regenerative efficacy measured as horizontal bone gain. Main study characteristics are displayed in Table 1. 

**Figure 3:**
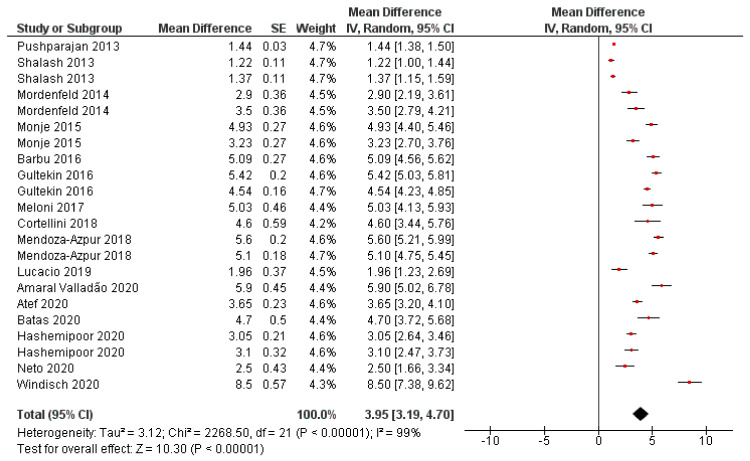
Horizontal bone gain forest plot. Weighted mean is presented at CI 95%. Heterogeneity was determined using Higgins (I2). A random-effects model was applied. Statistical significance was set at 0.05.

**Figure 4:**
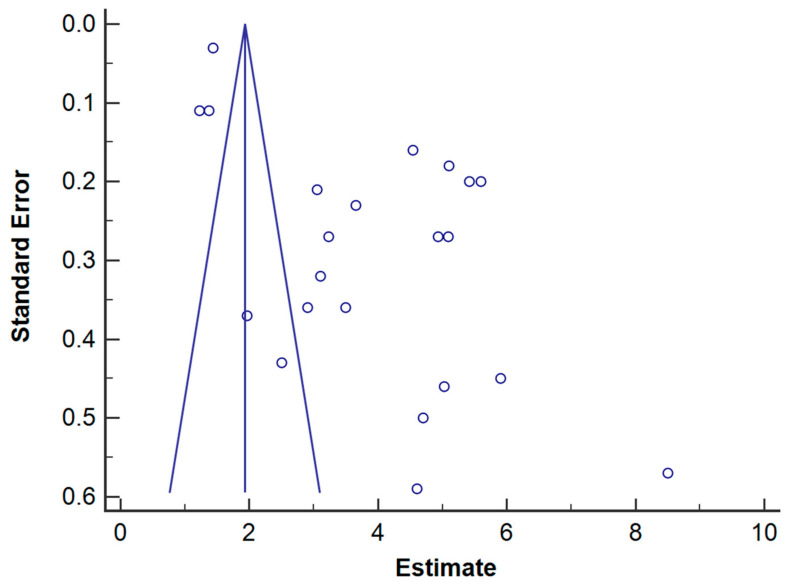
Horizontal bone gain funnel plot. Estimate of bone gain measurement is on the horizontal axis and study precision (standard error) appears on the vertical axis.

The mean of horizontal bone gain was 3.95 mm, ranging from 3.19 to 4.70 mm (CI 95%). Heterogeneity is I2 = 99% (CI 95%) and significance of the random-effects model was P < 0.001. Bone gain forest plot graph is displayed in Figure 3. Systematic heterogeneity is displayed at the funnel plot graph (Figure 4). 

The complications rate was 8.4%, while five studies did not report any type [16,19,24,25,27]. The 11 remaining studies demonstrate some clinical complications, including: membrane exposure, the most frequent (15 membranes exposures in seven studies [17,21,23,26,28,29,30]); nine dehiscences were reported in three studies [18,20,22]; two manuscripts referred to one infection each [23,26] (Table 1).

## 4. Discussion

The aim of this systematic review and meta-analysis was to obtain the most reliable scientific information regarding the efficacy of bone augmentation procedures in terms of bone gain in cases of horizontal and/or vertical ridge bone deficiencies, when using polymeric membranes for GBR. A great variability of results, measured as bone gain, does exist. Therefore, in this study it is intended to reduce heterogeneity of primary outcomes. For this purpose, only studies that counted with CBCT measurements were included in the review. It has been previously reported that there was variability between measurements performed at CBCT images and direct clinical measuring [31]. The follow-up of the patients included in the present review was set as between 3 and 7 months, and always before implant placement, in order to ensure that GBR processes were not influenced by the implants’ outcome. 

The first CBCT device (NewTom-9000; Quantitative Radiology, Verona, Italy) was described in 1998. Since then, a number of CBCT machines have been introduced into the market. The cost-effective technology of CBCT led to a speedy ingress into the field of dentistry with demand for commitment of dental professionals and dental educators to explore the applications of CBCT technology [32]. Nevertheless, CBCT is not employed in postsurgical assessments of bone grafts’ and implants’ position planning until 2006 [33,34,35]. It is the reason why, although a time frame restriction of 15 years was applied, the first published clinical trial using CBCT for bone augmentation evaluation was not found until 2013, which is the earliest study included in this review.

This systematic review was not limited to clinical trials to achieve more data about the use of polymeric membranes in GBR procedures. Sixteen studies were included, from which only four were randomized clinical trials. Case series, prospective, and retrospective designs were also included to achieve more data about the GBRs. In total, 381 GBRs have been analyzed, involving the mandible and the maxilla. Fourteen studies evaluated just horizontal bone gain after the augmentation surgery whereas two of them studied horizontal and vertical bone gain. Absorbable membranes were the most used (14 studies). Only three out of the 16 included studies tested non-resorbable membranes; two of them used a titanium reinforced dense polytetrafluroethyelene (Ti-d-PTFE) membrane and one with no reinforcing (d-PTFE). When using absorbable membranes, collagen membranes were the most frequently placed (93% of the studies using absorbable membranes), whereas only one used a commercialized pericardium membrane. The polymeric resorbable collagen membrane is the most employed for GBR procedures, having the higher number of published clinical studies [36]. Main advantages include easy manipulation, weak immunogenicity, a direct effect on bone formation and chemotaxis of gingival and periodontal ligament fibroblasts [37,38]. However, their rapid biodegradation by the enzymatic activity of macrophages and polymorphonuclear leucocytes or bacterial collagenases is their major drawback [39]. Then, the potential of losing space maintenance ability in physiological conditions is high and clinical results may be sometimes unpredictable [36]. 

Following the main results of the present research, GBR techniques using polymeric membranes may facilitate a horizontal bone gain from 3.19 to 4.70 mm at the alveolar ridge. It is a clinically relevant amount of bone, if we consider that placed implant may usually be from 3.5 to 4.5 mm in diameter, and a minimum of 1.5 mm of remnant bone is required around the placed implants [2]. Therefore, it may be speculated that the achieved horizontal volume after tested GBR techniques, should allow for implant placement with success in most of the clinical cases.

A bone gain of 8.5 ± 2.4 mm was described in the study that reported the highest horizontal bone gain [26]. It is a case series study in which a Ti-d-PTFE and bovine-derived xenograft in combination with autogenous bone chips. There are not many other studies in which non-resorbable membranes are used to treat horizontal bone defects. It may be that non-resorbable membranes requiring a second surgery and with complications derived from membranes exposition and contamination are preferred when treating vertical defects, in which procedures and healing time is longer and mechanical properties of membranes are a crucial prerequisite [40]. 

Ten of the selected studies used autologous bone + xenogenic as bone substitute [16,18,19,20,21,23,24,25,26,30], from which, 70% used particulate autologous bone + xenograft [16,18,21,24,25,26,30], 20% used autologous bone block [19,23], and one study compared both techniques [20], concluding that mean horizontal bone gain and width after healing were significantly greater in the group of autologous particulate bone compared to bone block. 

High bone gain results (5.9 ± 2.4 mm [25], 5.42 ± 0.76 mm [20], and 5.03 ± 2.15 mm [21]) were obtained in three studies which employed a common protocol. They used a collagen membrane with bovine-derived xenograft in combination with autogenous bone chips as bone filler. The highest clinical success when applying this bone combination was previously reported in one study published in 2019 [2]. It may be explained by the fact that an inorganic xenogenous graft could slow down the resorption of autogenous bone and also increase the volume to the grafted area [2].

The encountered rate of complications, 8.4%, is within the rate of other previous meta-analysis about bone regeneration procedures, ranging from 7.95% [2] to 22.7% [6]. Membrane exposure and dehiscences are also the most frequently reported complications in previous studies [2,6].

The attained total heterogeneity data between published studies is very high, 99% (95% CI) (Figure 3); it is also observable in the funnel plot graph (Figure 4). It may be explained by differences in implemented surgical techniques, employed biomaterials and operators. The surgical technique and execution are crucial for the success of bone augmentation procedures. Factors as achieving primary wound closure, adequate angiogenesis, space creation and maintenance, wound stability, membrane exposure, or microorganism colonization may influence the amount of bone regeneration that can occur [3]. The encountered systematic difference between studies may also be due to the small sample size of included studies (namely: ‘small-studies effect’) [41]. It should be considered that the experiments’ sample size ranges from 6 to 21 patients and from 6 to 29 surgical interventions. A mean bone gain of 5.9 mm (SD: 2.4 mm) was achieved in the study with the greatest sample size [25], a case series in which a combination of demineralized bovine bone matrix particles with autogenous bone, adding leukocytes and platelet rich fibrin was used. However, it should be remarkable that a high statistical significance was obtained at the random-model effects (*p* < 0.001).

Another meta-analysis has been previously published about bone regeneration at the alveolar ridge [6]. Differing from the present study, this study just considered GBRs performed simultaneously with dental implant placement. This surgical strategy is beneficial in terms of reducing the number of interventions. However, it usually negatively affects the total bone gain and increases the complications ratio. Membrane exposure was found in about 23% of the performed GBRs [6]. In this case, the most often used type of intervention was also a xenogeneic particulated grafting material and a resorbable collagen membrane. A mean bone gain of 4.44 mm (ranging from 0.11 to 7.72 mm) was obtained in a systematic review [2], which also studied horizontal bone ridge augmentation procedures but only applying xenogenous graft. It is a value slightly higher than the one obtained in the present research, but with a high standard deviation. Therefore, the present study was the only one considering several bone graft types, which is not reported in any other study. It is considered highly valuable for clinicians and researchers.

This systematic review and meta-analysis possess some strengths which differentiates it from previously published reviews. The registration of the research design in PROSPERO, prior to the beginning of the search, warrants that it has been shown to be associated with increased review quality [42]. Strict inclusion criteria, such as the need for a previous and follow-up CBCT and the exclusion of all the studies with clinical conditions that could alter the results such as immediate implant placement, simultaneal sinus lift, or socket preservation make the results more reliable. In addition, a thorough and rigorous analysis of the risk of bias and methodological quality of the studies included was carried out. As a result of all these methodological premises, a high level of significance (*p* < 0.001) was obtained, even when the random-effects model was applied. 

However, the study does not lack of certain limitations. The narrow focus of the question of systematic reviews is a crucial drawback of systematic reviews in general, since they do not allow for complex literature coverage. Apart from this, some of the differences encountered between the clinical trials could have been caused by the small-studies effect, due to their small sample sizes [41]. 

Additionally, as future perspectives, clinical researchers should try to perform more protocolized and randomized clinical trials in this area, since there is an enormous methodological and clinical heterogeneity in the identified studies. 

## 5. Conclusions

Through this systematic review and meta-analysis, we have been able to conclude that the existing scientific evidence suggests that GBR surgical procedure using polymeric membranes is a predictable technique, in order to achieve horizontal bone augmentation and, usually, the postoperative elapses with no complications. Clinicians can expect to reduce the horizontal bony defect from 3.19 to 4.70 mm, thus, permitting, in most of the cases, proper further implant placement. 

## Figures and Tables

**Table 1:** General overview of the included studies, investigating the outcomes of bone defects treated by guided bone regeneration (GBR) with bone graft materials (polymeric membranes and bone graft substitutes as autografts, xenografts, alloplasts, or allografts).

Author	Study Design	Patients GBRs	Bone Substitute	Membrane	Follow-Up Time	BG (mm) Mean (SD)	Complications
Pushparajan et al., 2013 [16]	CCT	10 patients 10 GBRs	DBBM particles Autogenous	Collagen	6 months	1.44 (0.09)	Not reported
Shalash et al., 2013 [17]	CCT	10 patients 10 GBRs	β-TCP particles	d-PTFE	6 months	1.22 (0.35)	2 membrane exposures
		10 patients 10 GBRs	β-TCP particles DBBM particles	d-PTFE		1.37 (0.35)	
Mordenfeld et al., 2014 [18]	RCT	13 patients 13 GBRs	DBBM particles (90) Autogenous (10)	Collagen	7.5 months	2.9 (1.3)	7 dehiscences
		13 patients 13 GBRs	DBBM particles (60) Autogenous (40)	Collagen		3.5 (1.3)	
Monje et al., 2015 [19]	CS	6 patients 9 GBRs	Illiac crest block DBBM particles	Collagen	5 months	4.93 (0.65)	Not reported
		8 patients 10 GBRs	Mandib ramus block DBBM particles	Collagen		3.23 (0.76)	
Barbu et al., 2016 [1]	RCT	11 patients 11 GBRs	Mandib ramus block DBBM particles + Autogenous	Pericardium	4 months	5.10 (0. 91)	3 patients with pain in donor site
Gultekin et al., 2016 [20]	CCT	12 patients 15 GBRs	DBBM particles Autogenous	Collagen	4–7 months	5.42 (0.76)	1 dehiscence
		12 patients 13 GBRs	Mandib ramus block DBBM particles	Collagen		4.54 (0.59)	
Meloni et al., 2017 [21]	CCT	18 patients 22 GBRs	DBBM particles Autogenous	Collagen	7 months	5.03 (2.15)	3 membrane exposures
Cortellini et al., 2018 [22]	CCT	10 patients 15 GBRs	L-PRF + DBBM particles	Collagen	5–8 months	4.6 (2.3)	1 dehiscence
Mendoza-Azpur et al., 2019 [23]	RCT	20 patients 20 GBRs	DBBM particles Autogenous	Collagen	6 months	5.6 (0.89)	6 membrane exposures 1 infection
		19 patients 19 GBRs	Mandib ramus block DBBM particles	Collagen		5.10 (0.77)	
Lucaciu et al., 2019 [24]	CS	13 patients 20 GBRs	ABBM particles Autogenous	Collagen	4 months	1.96 (1.64)	Not reported
Amaral Valladão et al., 2020 [25]	CS	18 patients 29 GBRs	L-PRF DBBM particles Autogenous	Collagen	7.5–8.5 months	5.9 (2.4)	Not reported
Atef et al., 2020 [26]	RCT	10 patients 10 GBRs	ABBM particles Autogenous	Collagen	6 months	3.65 (1.04)	1 membrane exposure 1 infection
Batas et al., 2020 [27]	CS	6 patients 6 GBRs	Allogenic bone DBBM particles	Collagen	5 months	4.7 (1.22)	Not reported
Hashemipoor et al., 2020 [28]	RCT	21 patients 21 GBRs	FDBA	Collagen	6 months	3.05 (0.98)	1 membrane exposure
		19 patients 19 GBRs	FDBA Autogenous	Collagen		3.10 (1.4)	
Neto et al., 2020 [29]	CCT	18 patients 22 GBRs	DBBM particles	Collagen	6–8 months	2.5 (2.02)	1 membrane exposure
Windisch et al., 2020 [30]	CS	15 patients 18 GBRs	DBBM particles Autogenous	d-PTFE	9 months	8.5 (2.4)	1 membrane exposure

* GBRs: Guided Bone Regeneration procedures; BG: Bone gain; CCT: Cohort and Case-Control Trial, RCT: Randomized Clinical Trial; CS: Case series; DBBM: Demineralized Bovine Bone Matrix; β-TCP: β-tricalcium phosphate; ABBM: Anorganic Bovine Bone Matrix; L-PRF: Leukocyte and Platelet Rich Fibrin; FDBA: Freeze Dried Bone Allograft.

## Data Availability

The data presented in this study are available on request from the corresponding author.
